# The probability of being identified as an outlier with commonly used funnel plot control limits for the standardised mortality ratio

**DOI:** 10.1186/1471-2288-12-98

**Published:** 2012-07-16

**Authors:** Sarah E Seaton, Bradley N Manktelow

**Affiliations:** 1 Department of Health Sciences, University of Leicester, 22-28 Princess Road West, Leicester, LE1 6TP, UK

**Keywords:** Funnel plot, SMR, Poisson, Probability, Outlier

## Abstract

**Background:**

Emphasis is increasingly being placed on the monitoring of clinical outcomes for health care providers. Funnel plots have become an increasingly popular graphical methodology used to identify potential outliers. It is assumed that a provider only displaying expected random variation (i.e. ‘in-control’) will fall outside a control limit with a known probability. In reality, the discrete count nature of these data, and the differing methods, can lead to true probabilities quite different from the nominal value. This paper investigates the true probability of an ‘in control’ provider falling outside control limits for the Standardised Mortality Ratio (SMR).

**Methods:**

The true probabilities of an ‘in control’ provider falling outside control limits for the SMR were calculated and compared for three commonly used limits: Wald confidence interval; ‘exact’ confidence interval; probability-based prediction interval.

**Results:**

The probability of falling above the upper limit, or below the lower limit, often varied greatly from the nominal value. This was particularly apparent when there were a small number of expected events: for expected events ≤50 the median probability of an ‘in-control’ provider falling above the upper 95% limit was 0.0301 (Wald), 0.0121 (‘exact’), 0.0201 (prediction).

**Conclusions:**

It is important to understand the properties and probability of being identified as an outlier by each of these different methods to aid the correct identification of poorly performing health care providers. The limits obtained using probability-based prediction limits have the most intuitive interpretation and their properties can be defined a priori. Funnel plot control limits for the SMR should not be based on confidence intervals.

## Background

The measuring and reporting of clinical outcomes has a long history [1]. However, over the last 20 years interest in reporting such outcomes has grown enormously. For example, in the foreword to the recent UK Government White Paper “Equity and excellence: Liberating the NHS” was the promise that:

*“… there will be a relentless focus on clinical outcomes. Success will be measured, not through bureaucratic process targets, but against results that really matter to patients – such as improving cancer and stroke survival rates*[2]*.”*

The measuring, monitoring and comparing of “success” requires robust methodologies and an understanding of the performance of these methods.

Clinical outcomes are often discrete counts, for example the number of deaths or the number of patients experiencing post-operative complications. The standardised mortality ratio (SMR) is commonly used as the measure for quantifying institution performance. The SMR is defined as the ratio of the observed number of events, for example deaths, to the expected number estimated using a reference population. It can usually be assumed that the observed number of events is an observation from a Poisson distribution [3].

Over recent years the use of the funnel plot to display SMRs graphically has been advocated as the standard method for institutional comparisons using cross-sectional data [4-9]. In the UK the use of funnel plots has been recommended by groups including the National Clinical Audit Advisory Group and the Association of Public Health Observatories [10,11]. With its roots in Statistical Process Control (SPC), a funnel plot comprises the plotting of an outcomes summary statistic from each individual institution against a specified ‘target’, together with upper and lower control limits. In the case of SMRs, the ‘target’ is the point at which the SMR equals one, i.e. the observed number of deaths equals the expected number. Usually two sets of control limits are displayed: typically 95% ‘alarm’ limits and 99.8% ‘action’ limits which roughly equate to ±2 and ±3 standard deviations respectively. Institutions falling outside the control limits are seen as potential outliers and it is recommended that possible causes for this are investigated [10]. Being identified as a potential outlier can have important consequences for the institution involved so it is crucial that funnel plots are produced and interpreted correctly.

Several methods have been suggested to estimate the control limits for funnel plots for the SMR and these proposed approaches can be divided into two broad categories: *confidence intervals*[5,12-17], and probability-based *prediction intervals*[9,12].

Confidence intervals for the Poisson distribution can be approximated using the Normal distribution (the Wald interval). However, it is well recognised that such limits can perform poorly, in particular for small samples [18]. An alternative ‘exact’ method using the link between the Chi-square and Poisson distributions has been used to construct control limits for funnel plots [3,19]. However, a potential problem in using confidence intervals is that, for funnel plots, they do not provide the answer to the question being asked. A confidence interval is used to make inferences on the value of a parameter given an observed sample of data. Funnel plot control limits, on the other hand, are used to make inferences about a future observation given known values of the parameters.

Prediction intervals are an alternative to confidence intervals. Using prediction intervals, funnel plot control limits are calculated so that an observation from a provider with an underlying performance equal to the ‘target’ (i.e. ‘in control’) will fall above, or below, the control limits with a known probability. This probability can be calculated directly, in this case using the cumulative probability distribution of the Poisson distribution.

Both confidence and prediction intervals can be calculated using standard statistical software or by using tools which have been specifically developed [12,15,17]. However, despite the theoretical differences between these two types of intervals, whichever method is used the intervals on a funnel plot are generally interpreted as prediction intervals: that is, for an ‘in control’ (only displaying variation which is expected) provider the probability of the observed outcome falling within the interval is equal to the nominal significance of the interval. For example, such an observation would have a probability of 0.95 of falling within the limits of a 95% interval and hence for an equal tailed interval a probability of 0.025 of falling above the upper limit and a probability of 0.025 of falling below the lower limit. While confidence intervals by definition may not have these probability characteristics, they may offer a good approximation and have the added advantage of being familiar to users.

In addition, although funnel plots are now widely used and can be easily obtained, the properties of the control limits remain unclear no matter which method is used to calculate the control limits. It has long been noted that exact probability statements are impossible in the case of discrete probability distributions [20]. Observed outcomes from a Poisson distribution can only take integer values and, therefore, *exact* probability based prediction limits do not exist. For example, for a sample from a population following a Poisson distribution with a mean of 10 the probability of observing more than 16 events is 0.027 whereas the probability of observing more than 17 events is 0.014. It is not possible to specify an outcome for which the probability is *exactly* 0.025.

It is unclear what the true probability is of an ‘in control’ unit falling outside of the control limits for the SMR and hence being labelled as a potential outlier. This knowledge is important in order to be able to draw meaningful inferences from funnel plots. This paper investigates the true probability of an ‘in control’ provider falling outside control limits for different numbers of expected events for the SMR.

## Methods

The probability of an observation from an ‘in control’ institution falling outside 95% and 99.8% control limits for funnel plots of the SMR were calculated for three commonly used methods based on the Poisson distribution: Wald confidence intervals; ‘exact’ confidence intervals and probability based prediction intervals.

### Confidence intervals

The limits of the Wald confidence interval for the Poisson distribution with mean *λ* (where *λ* is the expected number of events for the SMR) are given by λ±z1−α2λ, where *z*_*a*_ denotes the *a*^th^ percentile from the standard Normal distribution.

For the ‘exact’ confidence intervals the lower limit is given by 12χ2λ,α22 and the upper by 12χ2λ+1,1−α22, where $\chi_{b,c}^{2}$ denotes the *b*^th^ lower percentile of the chi-square distribution with *c* degrees of freedom.

### Prediction intervals

Probability-based prediction intervals were also calculated. The observed outcome of a new observation from an ‘in control’ institution will be expected to lie below the lower limit (*L*) with probability no greater than α/2 and above the upper limit (*U*) of a 100(1-α)% control interval also with probability no greater than α/2. Hence, the lower control limit *L* can be defined as the smallest integer *x*_*L*_ such that P(X ≤ *x*_*L*_) ≥ α/2 and the upper control limit *U* defined as the largest integer *x*_*U*_ such that P(X ≥ *x*_*U*_) ≥ α/2, where X = (0,1,2,…).

### Statistical analysis

The 95% and 99.8% control limits were calculated for values of the mean *λ* for 1 ≤ *λ* ≤ 10,000 using the three methods described. These limits were plotted for values of *λ* from 1 to 50. As these control limits are derived for observations from populations assumed to follow a known probability distribution (Poisson) it is simple to calculate the true probability of a new ‘in control’ observation falling outside the limits directly from the Poisson cumulative probability distribution. The probabilities of an observation from an ‘in control’ institution falling above or below each of the three sets of limits were calculated and the median, minimum and maximum values tabulated for pre-specified ranges of *λ*.

The SAS/BASE software, Version 9.2 was used for all calculations and SAS/GRAPH software, Version 9.2 was used to produce the graphs.

## Results

**Figure 1:**
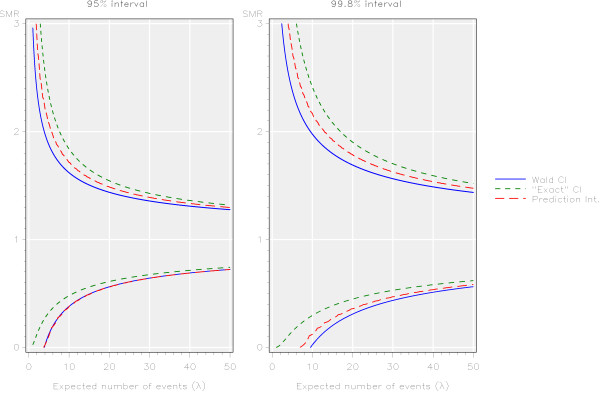
95% and 99.8% funnel plot control intervals for the SMR calculated by three different methods based on the Poisson distribution.

The control limits obtained using the three methods investigated in this paper were plotted for values of *λ* (where *λ* is the expected number of events) from 1 to 50 (Figure 1). The prediction interval has been smoothed using the interpolation method proposed by Spiegelhalter [6] for aesthetic purposes only. This interpolation method does not affect the probability of being identified as an outlier. No interpolation has been applied to the confidence interval methods. For both the 95% and 99.8% control limits the values for the limits varied greatly between the three methods. For both sets of control limits the values of both the lower and upper control limits obtained using the ‘exact’ confidence interval were higher than those of the other two methods. The values obtained using the Wald confidence interval method tended to be the lowest, although these were very similar to the values from the prediction interval for the lower 95% control limits.

However, since the SMR can only take certain values at each value of *λ*, due to the fact that the observed number of events must be a whole number, the actual probability of an observation falling outside of a limit can be the same even if the values of the limits themselves are different. So the probability of an observation from an ‘in control’ institution falling below the lower limit or above the upper limit of two-sided 95% and 99.8% control limits was calculated directly from the cumulative Poisson distribution for values of *λ* up to *λ* = 10,000. The range and median value of these probabilities was calculated for intervals of *λ* (Table 1).

**Table 1:** Median, minimum and maximum probability of an observation from an ‘in control’ process falling below the lower limit, and above the upper limit, of 95% and 99.8% funnel plot control limits for the SMR using three different methods to calculate the limits

	**Wald confidence interval**			**‘Exact’ confidence interval**			**Prediction interval**		
***λ***	**Median**	**Min**	**Max**	**Median**	**Min**	**Max**	**Median**	**Min**	**Max**
***95% control limits***									
***Lower***									
1-50	0.0184	0	0.0250	0.0325	0.0219	0.3679	0.0186	0	0.0250
>50-100	0.0216	0.0172	0.0251	0.0287	0.0248	0.0351	0.0215	0.0172	0.0250
>100-500	0.0233	0.0194	0.0251	0.0267	0.0248	0.0317	0.0233	0.0194	0.0250
>500-1000	0.0240	0.0225	0.0251	0.0261	0.0249	0.0277	0.0239	0.0224	0.0250
>1000-10000	0.0246	0.0232	0.0250	0.0254	0.0250	0.0269	0.0246	0.0232	0.0250
***Upper***									
1-50	0.0301	0.0203	0.0841	0.0121	0.0003	0.0177	0.0201	0.0052	0.0250
>50-100	0.0280	0.0247	0.0331	0.0166	0.0129	0.0196	0.0220	0.0184	0.0250
>100-500	0.0266	0.0248	0.0307	0.0203	0.0157	0.0225	0.0234	0.0200	0.0250
>500-1000	0.0260	0.0249	0.0275	0.0220	0.0203	0.0232	0.0240	0.0226	0.0250
>1000-10000	0.0254	0.0249	0.0268	0.0238	0.0216	0.0244	0.0246	0.0233	0.0250
***99.8% control limits***									
***Lower***									
1-50	0.0002	0	0.0006	0.0028	0.0016	0.3679	0.0006	0	0.0010
>50-100	0.0005	0.0003	0.0007	0.0017	0.0014	0.0026	0.0008	0.0006	0.0010
>100-500	0.0007	0.0005	0.0009	0.0013	0.0012	0.0020	0.0009	0.0007	0.0010
>500-1000	0.0008	0.0007	0.0009	0.0012	0.0011	0.0013	0.0009	0.0009	0.0010
>1000-10000	0.0009	0.0008	0.0010	0.0011	0.0010	0.0012	0.0010	0.0009	0.0010
***Upper***									
1-50	0.0021	0.0014	0.0133	0.0002	0.0000	0.0004	0.0007	0.0002	0.0010
>50-100	0.0016	0.0013	0.0021	0.0004	0.0002	0.0005	0.0008	0.0007	0.0010
>100-500	0.0013	0.0011	0.0018	0.0006	0.0004	0.0007	0.0009	0.0007	0.0010
>500-1000	0.0011	0.0011	0.0013	0.0007	0.0006	0.0008	0.0009	0.0009	0.0010
>1000-10000	0.0011	0.0010	0.0012	0.0009	0.0007	0.0009	0.0010	0.0009	0.0010

The probabilities here are calculated directly from the cumulative Poisson distribution.

### Wald confidence intervals

**Figure 2:**
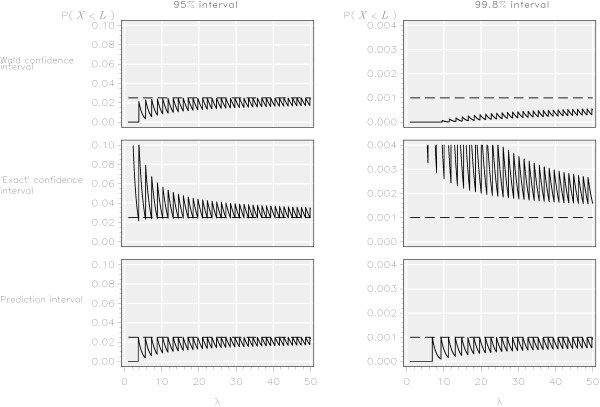
Probability of an observation from an ‘in control’ process falling below the lower limit of 95% and 99.8% funnel plot control intervals for three methods based on the Poisson distribution.

For both the 95% and 99.8% control limits obtained using Wald confidence intervals the probability of an observation from an ‘in control’ institution falling below the lower limit was almost always less than the nominal probabilities of 0.0250 and 0.001 (Figure 2) for the values of *λ* investigated. Although the probabilities tended to the nominal values as *λ* increased, even for 500 ≤ *λ* ≤ 1,000 the median probability was 0.0240 (range 0.0225 to 0.0251) for the 95% interval and 0.0008 (range 0.0007 to 0.0009) for the 99.8% interval (Table 1).

**Figure 3:**
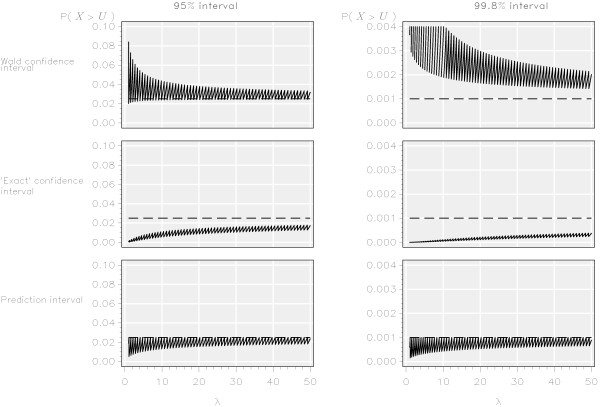
Probability of an observation from an ‘in control’ process falling above the upper limit of 95% and 99.8% funnel plot control intervals for three methods based on the Poisson distribution.

Conversely, the probability of an observation falling above the upper control limit by the Wald confidence interval was generally greater than the nominal probabilities of 0.025 and 0.001 (Figure 3). For values 500 ≤ *λ* ≤ 1,000 the median probability was 0.0260 (range 0.0249 to 0.0275) for the 95% interval and 0.0011(range 0.0011 to 0.0013) for the 99.8% interval (Table 1).

### ‘Exact’ confidence interval

The probability of an observation from an ‘in control’ institution falling below the lower control limits obtained using ‘exact’ confidence intervals was always greater than the nominal probability (Figure 2). For 500 ≤ *λ* ≤ 1,000 the median probability was 0.0261 (range 0.0249 to 0.0277) for the 95% interval and 0.0012 (range 0.0011 to 0.0013) for the 99.8% interval (Table 1).

However, the coverage probability of falling above the upper limit of the ‘exact’ confidence interval was generally less than the nominal probabilities of 0.025 and 0.001 (Figure 3). Although the probabilities tended to the nominal values as *λ* increased, even for values 500 ≤ *λ* ≤ 1,000 the median probability was 0.0220 (range 0.0203 to 0.0232) for the 95% interval and 0.0007 (range 0.0006 to 0.0008) for the 99.8% interval (Table 1).

### Prediction intervals

By definition, the probability of an observation from an ‘in control’ institution falling outside the control limits was always less than the nominal probabilities of 0.025 and 0.001 (Figures 2 and 3) for both the lower and upper control limits obtained from the prediction interval. As with the other methods, the probability of an observation from an ‘in control’ provider lying outside of the limits tended to the nominal values as *λ* increased. For 500 ≤ *λ* ≤ 1,000 the median probability was 0.0239 (range 0.0224 to 0.0250) for the 95% interval and 0.0009 (range 0.0009 to 0.0010) for the lower control limit of the 99.8% interval (Table 1).

## Discussion

Funnel plots are now commonly used tools for the identification of health care providers with potentially outlying performance. In the case of the SMR, funnel plots have the convenience of allowing the SMR from an individual provider to be plotted on a graph where the control limits have been pre-drawn. Their interpretation is, at first sight, also straightforward: the observed SMR for a provider whose underlying performance matches the ‘target’ will fall outside the control limits with a known (nominal) probability. However, as has been shown in this paper the true probability of falling outside of the limits does not always match this nominal value. Two reasons for this mismatch were investigated here: 1) the use of different methods to construct the control limits; 2) the effect of discrete outcomes in preventing the specification of exact probabilities.

Three commonly used methods based on the Poisson distribution have been investigated for 95% and 99.8% control limits for funnel plots of the SMR. Two of these methods were based on confidence intervals and the third was the prediction interval derived using the Poisson cumulative probability distribution. The methods produced different control limits and different probabilities for an ‘in control’ unit to fall outside of these limits. The probability of a provider being identified as a potential outlier is dependent, therefore, on the method used to calculate the control limits.

Whilst no one method performs well for all values of *λ* (the expected number of events), the ‘exact’ confidence interval method performed particularly poorly and should be avoided if a probability close to the nominal value is desired. The probability of the observed outcome from an ‘in control’ institution falling outside of the limits of ‘exact’ confidence interval can be quite different from the assumed nominal values. For example, if the expected number of events is between 1 and 50 the median probability of an ‘in control’ institution falling above the upper limit of a 95% control interval and, hence, being identified as a potential outlier, is 0.012 instead of 0.025: i.e. less than half the presumed probability. Often with SMRs very small numbers of events occur and, therefore, the potential for being identified by this method is decreased particularly when *λ* is small.

It is also important to consider the properties of the method used when interpreting any results or limits produced. Although confidence intervals are often more familiar to the reader, a disadvantage of their use in this context is that they are often interpreted incorrectly.

Probability-based prediction intervals allow a more straightforward interpretation of control limits. In this paper they were defined so that the probability of falling outside a control limit was always less than, or equal to, the nominal probability: for example, the probability of falling above the upper limit of 95% control limits is always less than, or equal to, 0.025. However, the control limits could equally have been derived so that the probability of an observation from an ‘in control’ provider falling outside of the limits was *at least* equal to the nominal value or, indeed, some combination of the two approaches to obtain a value that produced a probability closest to the nominal value [6,21,22]. The decision of which of these options to use will depend on various factors, including the clinical question of interest. However, the important point is that if the control limits are obtained from probability-based prediction intervals then this property of the limits can be specified a priori. This cannot be done if the control limits are based on confidence intervals.

Funnel plots can be used to answer questions other than just “Which providers’ results are not compatible with the target” [23]. While investigating alternative approaches is beyond the scope of this paper, the same principle applies that only the use of prediction intervals can produce control limits with probability properties specified a priori.

It also seems appropriate that there is a need for the limits to be symmetrical, that is have the same properties for falling above the upper control limit as falling below the lower control limit. The Stata function FUNNELCOMPAR, for example, has asymmetrical tails in that the probability of an observation falling below the lower limit is always less than, or equal to, the nominal value (i.e. P(X ≤ *x*_*L*_) ≤ α/2) whereas the probability of an observation falling above the upper limit is always at least the nominal value (i.e. P(X ≥ *x*_*U*_) ≥ α/2)[12]. Such asymmetry makes the funnel plots difficult to interpret.

It could be argued that any control limits are always only approximate given the uncertainties in the data, any statistical modelling, the target, etc. However, funnel plot limits continue to be used for identification of potentially poorly performing institutions in order to initiate further investigations. Therefore a full and correct understanding of funnel plots is needed in order to avoid the unnecessary investigation of ‘in control’ providers or the failure to investigate the true outliers. Such investigations can have important consequences in themselves whether the provider is ultimately deemed to be a true outlier or not.

In this paper 95% and 99.8% control limits were investigated as these are the limits most commonly used for monitoring health care providers. These particular control limits are unlikely to be optimal in all circumstances and careful consideration should always be given to the choice of limits. However, the properties of the potential methods to calculate the limits described in this paper are likely to hold whatever limits are selected.

## Conclusions

This paper has described the true probability of an ‘in control’ institution being classed as a potential outlier using funnel plot control limits for the SMR obtained by three commonly used methodologies. The control limits obtained using probability-based prediction limits have the most logical and intuitive interpretation and their properties can be defined a priori. Funnel plot control limits for the SMR should not be based on confidence intervals.

## Competing interests

The authors declare that they have no competing interest.

## Authors’ contributions

BM developed the initial concept for this work. Both authors performed the statistical analyses, contributed to writing of the paper and agreed the final draft.

## Pre-publication history

The pre-publication history for this paper can be accessed here:

http://www.biomedcentral.com/1471-2288/12/98/prepub
